# Direct observation of killer whales preying on white sharks and evidence of a flight response

**DOI:** 10.1002/ecy.3875

**Published:** 2022-11-27

**Authors:** Alison V. Towner, Alison A. Kock, Christiaan Stopforth, David Hurwitz, Simon H. Elwen

**Affiliations:** ^1^ Department of Ichthyology and Fisheries Science Rhodes University Makhanda South Africa; ^2^ Dyer Island Conservation Trust Kleinbaai South Africa; ^3^ South African National Parks Cape Research Centre Cape Town South Africa; ^4^ South African Institute for Aquatic Biodiversity (SAIAB) Makhanda South Africa; ^5^ Drone Fanatics SA Mossel Bay South Africa; ^6^ Simons Town Boat Company Simon's Town South Africa; ^7^ Sea Search Research and Conservation NPC Cape Town South Africa; ^8^ Department of Botany and Zoology, Faculty of Science Stellenbosch University Stellenbosch South Africa

**Keywords:** cultural transmission, ecology of fear, human–wildlife conflict, livelihood impacts, predator–prey interactions

Killer whales (*Orcinus orca*) and white sharks (*Carcharodon carcharias*) are marine apex predators that shape prey behavior and even entire ecosystems through direct predation effects and indirect fear effects (e.g., Estes et al., 2009; Heithaus et al., 2008). Killer whales are known to occasionally hunt white sharks, although only three studies have formally described this behavior and the associated flight responses in white sharks (Jorgensen et al., 2019; Pyle et al., 1999; Towner, Watson, et al., 2022). However, direct observation of predation has been lacking, giving rise to speculation on the hunting strategies used by killer whales to capture and kill white sharks and the subsequent behavioral impact on surviving white sharks in the area where the predation took place, including the cues and timing of shark responses to these events.

On 16 May 2022 at 14:36 PM, author CS, a hobbyist drone pilot, filmed a group of killer whales at Hartenbos Beach and river mouth, Mossel Bay, South Africa (34°07′ S, 22°07′ E) using a private drone (DJI Mavic Air^2^S with a 1″ CMOS sensor and 20 MP camera). Five killer whales were observed for around 40 min, and the drone followed them for a total of 30 min. Owing to drone memory and battery constraints, only the two segments described here were recorded. Initially, the group of five killer whales were together but separated as direct observations began, with KW1 (“Starboard,” a male killer whale implicated in earlier shark predations in South Africa, Figure 1j inset) and KW2 (unidentified) began moving ~500 m east toward the Hartenbos River mouth, a known high‐use area for white sharks (Jewell et al., 2013). The drone first flew over three of the five killer whales, ~400 m off Hartenbos beach and started recording. The following event was filmed as a single clip with all three whales continuously visible in the frame: KW3 and KW4 were observed swimming close together on the surface but facing opposite directions. After 7 s KW5 appeared from depth between KW3 and KW4 and used its rostrum to push an estimated ~3 m total length white shark to the surface from the ventral side, while rolling the shark to its right side (Figure 1a). The shark appeared dead as KW5 brought it up to the surface, with the shark's white underbelly partially visible (Figure 1b). KW5 bit into the shark, in the region of the pectoral fins, and a large cloud of blood appeared (Figure 1c). KW5 then dove down with the shark still in its mouth but released it and began to move off when KW3 rapidly approached and made a very close pass by the white sharks tail, appearing to grab the tail before diving down out of sight (Figure 1d). KW3 surfaced again away from the last observed location of the white shark; then all three KWs moved offshore to the west. At 15:17 PM, the drone then flew east to the river mouth and filmed KW1 and KW2, before they also moved in the same direction as KWs 3, 4, and 5. The group remained spread out and swam off toward Diaz Point, the western headland of the bay. No shark carcasses were observed or recovered on this or subsequent weeks.

**FIGURE 1:**
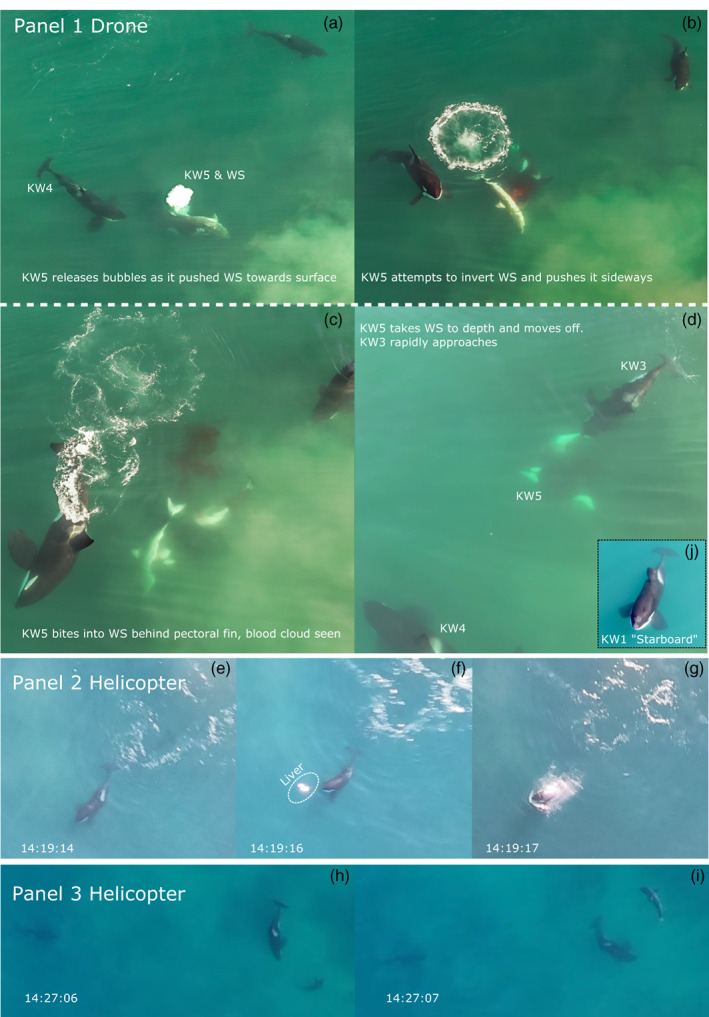
Series of images taken off Hartenbos Beach, Mossel Bay, South Africa. Panel 1: screen grabs from video filmed on a drone by author CS showing first confirmed observation of a group of killer whales killing a white shark. Inset in (d) shows KW1, an animal with a bent dorsal fin known as “Starboard,” one of the pair of killer whales previously implicated in shark predations in South Africa, which was part of the group. Panel 2: Series of photographs taken on a mobile phone from a helicopter (D. Archer, Mossel Bay Helicopters) showing potential consumption of a free‐floating shark liver. Panel 3: Screen grabs from video take on mobile phone showing circling behavior of a white shark avoiding a killer whale while a second whale approaches.

On 16 May 2022, between 14:00 and 15:00 PM, D. Archer of Mossel Bay Helicopters flew a series of short tourist flights over the same area of Hartenbos (34°07′ S, 22°07′ E). The pilot witnessed two white sharks being killed by killer whales, but neither was fully captured on film. However, using a Samsung S21 cell phone, the pilot captured a series of images and short video clips, which partially captured the interactions between four of the previously described killer whales and several white sharks (Figure 1e,f,g and h,j). Given the overlap in time and area, we assumed it was the same group captured by the drone footage. Two video sequences at 14:07 and 14:27 PM showed two different killer whales (*Starboard* in the first clip) closely following large white sharks at <1 killer whale body length. In both clips, the sharks displayed evasive behaviors, circling back tightly with the whale following, but both moving slowly. In the later clip, a second killer whale is seen directly approaching the first killer whale and white shark to within five body lengths before visuals are lost. In these videos, one and two other white sharks swam <100 m from the killer whales without noticeable reactions. In another sequence, four different killer whales are seen at the surface, but only *Starboard* can be identified because of his collapsed dorsal fin. Between these video clips (at 14:19 PM), a series of three images over 4 s showed what appears to be the consumption of a free‐floating shark liver. The liver is roughly the size of the killer whale's head and appears at the surface before being taken into the killer whale's mouth.

Three lines of evidence suggest that white sharks fled the area immediately and remained away for at least 7 weeks (Figure 2). First, 4 min before the predation occurred, author CS and other beach‐based observers reported seeing white sharks fleeing the area in several directions, some swimming into extremely shallow water <2 m deep. Second, opportunistic drone flight data were available from 2 April to 7 July 2022 (52 flights; 1477 min of drone footage) from the same area. The average drone flight before the predation on the 16 May lasted 48 min, covered 12.4 km of coastline, and counted four different white sharks (range 1–8, Figure 2a). Based on the sharks' size, individuals in one frame and their location during the drone flight, 10 different sharks were observed during the 75 min of flight time on 16 May. During the 8 days after the predation event (eight drone flights, average flight duration of 59 min covering 6.6 km), only one shark was recorded, the day after the observed predation (Figure 2a). Third, commercial white shark cage diving operators, White Shark Africa, operating in Mossel Bay conducted 38 trips on 31 days between 6 May and 6 July 2022. They encountered an average of 3.3 individual sharks per trip (range 0.5–6 sharks per trip, Figure 2b) before the predations and 0 sharks for 45 days after the predation, when the first white shark was seen again on 1 July (Figure 2b). Four sharks (identified by their size and body patterns) were observed on the day of the predation. Finally, a 3.8‐m female white shark, fitted with a towed SPOT6 satellite tag (Wildlife Computers) in Mossel Bay on 25 April 2022, stayed in the area and was last detected in Hartenbos, Mossel Bay, on 14 May 2022 (2 days before the predation) and then again 400 km eastward at Algoa Bay on 25 May (A. Towner, unpublished data). Given the well‐documented, predictable year‐round presence of white sharks in Mossel Bay (Jewell et al., 2013; Ryklief et al., 2014), the sudden absence of white sharks for several weeks immediately after the predation event supports a flight response by surviving white sharks in the area.

**FIGURE 2:**
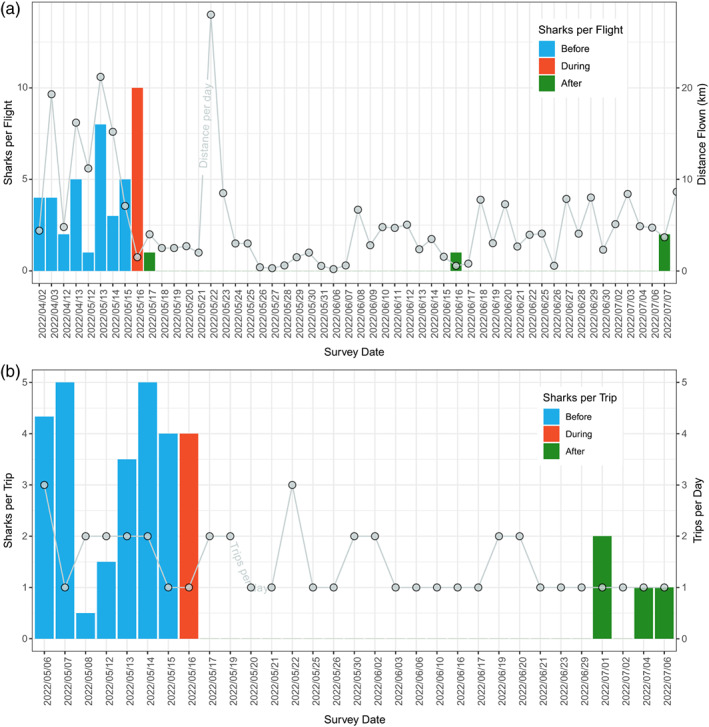
(a) Bars show number of white sharks observed per drone flight by author CS in Hartenbos, Mossel Bay area, before, during, and after predation event described here. Line shows total flight distance per day for drone from pilot log books. (b) Bars show average number of white sharks observed per trip by commercial cage diving operator working in Mossel Bay before, during, and after predation event described here. Line shows number of trips per day. Note that days without surveys are not included in either plot.

At least two, possibly three, white sharks were killed by a group of killer whales on 16 May in Mossel Bay over a period of around 71 min. Prior evidence of white shark deaths from killer whale predations was limited to recovered carcasses (Towner, Watson, et al., 2022). The combined footage and events described here provide novel insights into how killer whales cornered, captured, and incapacitated white sharks and how the sharks responded. In one case, a killer whale attempted to roll and invert a white shark into a position that would result in tonic immobility (Henningsen, 1994; Pyle et al., 1999) before biting into its abdomen just behind the pectoral fins. This confirms the strategy hypothesized from the examination of previous shark carcasses based on missing livers and bite marks on pectoral fins and torn pectoral girdles (Engelbrecht et al., 2019; Towner, Watson, et al., 2022). The consumption of what appears to be a large piece of drifting liver confirms that the liver is positively buoyant and may escape the torn open body cavity of the shark and float to the surface.

The videos also provided new insights into the evasive behavior employed by white sharks. Killer whales closely, but slowly, followed white sharks, which in turn displayed evasive action by tightly circling the killer whales while keeping them within sight. Similarly, Cape fur seals had a better chance of evading white sharks by staying close to them and keeping the shark within sight (Martin et al., 2005), and turtles successfully evaded tiger sharks by swimming in tight circles around the shark (Andrzejaczek et al., 2019). However, because killer whales are social and hunt in groups, this evasive tactic may not be effective. The energetic needs of an adult male killer whale (1394 Ml per day; Reisinger et al., 2011) are roughly equivalent to the energetic value of the liver of an adult ~428‐kg white shark (Pethybridge et al., 2014), and even small populations with specialized diets can have profound impacts on ecosystems (Estes et al., 2009). Although behavioral implications are beyond the scope of this study, we show that more killer whales are hunting white sharks in South Africa than just the two previously identified ones (Towner, Watson, et al., 2022). If cultural transmission between killer whales is occurring (Amelot et al., 2022), it will have wider‐reaching impacts on shark populations and will need to be considered in future studies.

## CONFLICT OF INTEREST

The authors declare no conflict of interest for any of this work.

## Data Availability

Drone and helicopter footage (Towner, Kock, et al., 2022) are available on Figshare at https://doi.org/10.6084/m9.figshare.20625222. Drone and helicopter survey data (Towner, 2022) are available on Figshare at https://doi.org/10.6084/m9.figshare.20533320.
